# Changes in Psychological States During Instrumented Biofeedback for Dyssynergic Defaecation and Fecal Incontinence Are Associated With Changes in Patient‐Reported Outcomes

**DOI:** 10.1111/nmo.70338

**Published:** 2026-05-10

**Authors:** Michael P. Jones, Yoav Mazor, Alissa Beath, Gillian Prott, Carol Sequeira, Allison Malcolm

**Affiliations:** ^1^ School of Psychological Sciences, Faculty of Medicine, Health and Human Sciences Macquarie University Sydney New South Wales Australia; ^2^ Neurogastroenterology Unit and Department of Gastroenterology Royal North Shore Hospital St Leonards New South Wales Australia; ^3^ Department of Gastroenterology Tel Aviv Medical Center Tel Aviv Israel; ^4^ School of Psychology The University of Sydney Sydney New South Wales Australia; ^5^ Faculty of Medicine and Health The University of Sydney Sydney New South Wales Australia

**Keywords:** biofeedback, dyssynergic defecation, fecal incontinence, psychological traits

## Abstract

**Background:**

Chronic constipation and fecal incontinence (FI) are severe and chronic conditions associated with reduced quality of life and distress for those affected. Instrumented anorectal biofeedback has demonstrated efficacy in both conditions and is often described as a learning process, suggesting efficacy is mediated via central cognitive and motivational pathways, but empirical evidence is lacking. The aim of this exploratory study was to seek evidence of change in psychological processes as central mediators of biofeedback treatment efficacy.

**Methods:**

Patients (*n* = 135) were prospectively recruited from a tertiary care hospital, diagnosed with constipation likely due to dyssynergic defecation and/or FI. A range of psychological traits covering cognitive processes, emotion regulation, and others were measured at the beginning and end of therapy, along with patient outcomes. Changes in psychological traits were correlated with corresponding changes in patient outcomes, including condition‐specific quality of life measures.

**Results:**

Improvement in constipation impact on quality of life was most notably correlated with reduced external locus of control (*p* = 0.004) and reduced use of acceptance as an emotion regulation strategy (*p* = 0.048). Correlates of improvement in FI impact on quality of life were also related to reduced use of acceptance as well as improvements in measures of executive function (*p* = 0.0003 to *p* = 0.03), cognitive flexibility (*p* = 0.0001 to *p* = 0.02), and emotional regulation (*p* = 0.002 to 0.045).

**Conclusions:**

Our data strongly support the hypothesis that improvements in the disease impact of constipation and FI during biofeedback are related to concomitant changes in central psychological processes, suggesting that psychological factors play an important part in the mechanism of action of biofeedback therapy.

## Plain Language Summary

Biofeedback therapy is a strongly evidence‐based therapy for patients with severe chronic constipation and/or fecal incontinence. However, empirical evidence for how biofeedback therapy operates to achieve improvement in patient outcomes is limited, and selection of patients for this resource‐intensive therapy is left largely up to the judgment of individual clinicians. This study sought to provide insights into central psychological processes that might be involved in its mechanism of action. This would both yield insights into how biofeedback operates and provide options for future tailoring of the therapeutic approach. The study provides clear empirical evidence that improvements in patient outcomes with instrumented biofeedback therapy for chronic constipation and fecal incontinence co‐occur with changes in psychological traits that were hypothesized to be related to central processes involved in anorectal sensation and control. An important role for aspects of patient emotion regulation and for executive function was identified.

## Graphical abstract

Central psychological processes play an important role in biofeedback success.

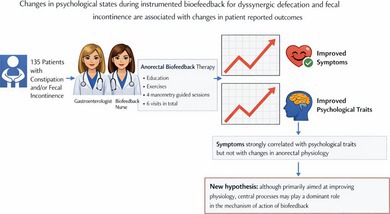

## 1 Introduction

Chronic constipation and fecal incontinence are very common conditions associated with anxiety and depression, significantly reduced in quality‐of‐life and leading to high health care utilization [1, 2, 3]. Although causes of chronic constipation are numerous [4], defecatory disorders are involved in at least one third [5] and pathophysiologies include paradoxical contraction of anal and pelvic floor muscles [6] and inadequate rectal pressure being exerted to expel faeces [7]. Fecal incontinence, on the other hand, may result from inadequate pressure in the anal sphincter mechanism to contain the rectal content. Disorders of sensation may contribute to both [8].

Conservative treatment approaches for both chronic constipation due to dyssynergic symptoms (DS) and fecal incontinence (FI), utilizing lifestyle measures and therapies that influence stool form and transit, may be ineffective in a proportion of cases [9, 10]. In this situation, anorectal biofeedback (which focuses on normalizing physiology) has been recommended, with convincing data to support its use in both conditions [11]. Biofeedback does not, however, achieve complete success in all patients. Patient and treatment features which are associated with patient outcomes of biofeedback therapy have been sought [9, 12, 13] but with limited success in identifying traits which reliably and causally mediate the outcome of treatment. This may reflect some lack of specificity in our understanding of the mechanism by which biofeedback operates. Furthermore, there is often a disconnect between improvements seen in overall patient‐reported symptoms compared to changes in objective physiology [10, 14] despite the main focus of biofeedback being to correct disordered physiology.

A relatively unexplored area of investigation into how patients benefit from biofeedback therapy is the analysis of neuropsychological changes that occur during therapy. In health, the physiological process of detecting the presence of stool in the rectum and the coordination of the pelvic floor and external sphincter muscles to achieve defaecation and continence is well understood [15], as are the neurological pathways involved [14]. However, the cognitive, affective, and motivational processes involved in these coordinated activities are less explored and may be crucial to understanding the mechanistic action of biofeedback. Biofeedback therapy is often described as a learning process [10] through which patients learn to accurately recognize rectal filling and then coordinate the use of abdominophrenic, pelvic floor, and anal muscles to achieve optimum defecation and continence [16]. More specifically, biofeedback therapy is thought to be a form of operant conditioning [9] in which behaviors that result in positive outcomes are more likely to be repeated in the future. An influence of biofeedback on the autonomic nervous system of the colon has been suggested [17]; however, it is possible that psychological or central effects (such as cognitive load theory [18] and learning theory [19]) predominate. Psychological processes have been implicated in biofeedback therapies in different contexts [20]. Further, in psychotherapy, psychological processes such as cognitive flexibility [21] are known to be key mechanisms through which therapeutic change occurs, raising the question of whether similar processes may be involved in biofeedback training.

The purpose of this study is to evaluate whether any of a range of neuropsychological processes change in concert with changes in defecatory control after biofeedback therapy for constipation or FI. Specifically, we correlate the gradient of change in a range of psychological measures with the gradient of change in a panel of patient outcomes. A panel of outcomes was selected that taps into distinct aspects of the patient experience. While finding correlations would not prove a causal role for such processes, it would be strongly suggestive. Furthermore, we sought to evaluate whether such a change in neuropsychological processes correlates with patient outcomes.

## 2 Methods

### 2.1 Patients

Patients were recruited prospectively within a Neurogastroenterology unit at a tertiary referral hospital in Sydney, Australia. Clinical diagnoses were chronic constipation (CC) with a predominance of defecatory symptoms that were considered by the treating neurogastroenterologist to be due to dyssynergia or FI. Patients undertook high‐resolution water‐perfused anorectal manometry testing plus the rectal sensory test and balloon expulsion test largely in accordance with the London protocol [22]. Patients subsequently underwent a course of instrumented anorectal biofeedback therapy, which has been described previously [9, 23]. Ethics approval was obtained from the Northern Sydney Local Health District Human Research Ethics Committee (2019/ETH08482). Change from baseline (pre‐treatment) to end‐of‐therapy was evaluated. In a parallel study [9], utilizing the same sample, we address a different research question pertaining to whether psychological, cognitive, and emotional measures assessed pre‐treatment could be used to predict clinical outcomes of treatment.

### 2.2 Patient Outcomes

Outcome measures were selected according to the presenting condition. For those clinically diagnosed with CC, change from baseline to end‐of‐therapy in the disease‐specific constipation quality‐of‐life scale (PAC‐QoL [18]), as well as change in complete bowel motions (CBM) per week and complete bowel motions as a percentage of all bowel motions over the same period, were selected. Patients were not asked to cease laxative or prokinetic use, and hence, the spontaneity of bowel motions could not be assessed. The PAC‐QoL is scored such that higher values represent greater impact on quality of life. For patients presenting with FI, change from baseline to end‐of‐therapy in the fecal incontinence severity index [19] (FISI), the fecal incontinence quality‐of‐life scale [24] (FI‐QoL), and a ten‐point visual analogue scale (VAS) of perceived control over bowel movements were selected. The FI‐QoL is scored such that higher values represent a higher quality of life, and higher scores on the FISI represent greater severity. All psychological questionnaires were administered via an iPad in the waiting room at the time of consultation with the gastroenterologist at the beginning and end of the biofeedback program.

### 2.3 Psychological Measures

Potential psychological constructs whose change in parallel with changes in patient outcome were evaluated included perceived stress, via the perceived stress scale [25] (PSQ), cognitive processes, via the Behavior Rating Inventory of Executive Function [26] (BRIEF), Cognitive Flexibility Inventory [27] (CFI), and the Cognitive Emotion Regulation Questionnaire [28] (CERQ), locus of control, via the Multidimensional Health Locus of Control scale [29] (HLOC), treatment credibility/expectancy via the Credibility/Expectancy Questionnaire [30] (TE), generalized self‐efficacy [31] (GSES), and therapeutic alliance between patient and doctor via the Working Alliance Inventory [32] (WAI).

An explanation of what each of these measures, including subscales, means in tangible terms is provided in Table 1, and readers may find this useful to refer to when interpreting the results. It is useful to consider that some of the constructs utilized are generally considered beneficial, e.g., generalized self‐efficacy, working alliance, cognitive flexibility, and treatment credibility/expectancy, while others are generally considered detrimental, e.g., anxiety, depression, and perceived stress. Others, however, such as locus of control, are more context‐dependent. While there is reason to hypothesize that the psychological traits considered are related to change in patient outcome, there is not always reason to hypothesize about every specific domain of each trait. For this reason, and to minimize statistical type I error risk, only overall domain scores for each trait are used, unless there is a specific reason. Examples of the latter include emotion regulation, where some strategies may be viewed as adaptive (e.g., positive reappraisal) while others are generally maladaptive (e.g., catastrophizing).

**TABLE 1:** Psychological measures and their interpretation.

Construct name	Abbreviation	Meaning	Subscales	Score range and interpretation
Behavior Rating Inventory of Executive Function (Adults) [33]	BRIEF	Executive function allows people to manage complex or novel situations by coordinating thoughts and actions, regulating impulses, and adapting to changing demands	Only the overall score is used	Raw scores are converted to T‐scores (mean = 50 for each index and mean = 100 for composite score, SD = 10). Higher scores indicate poorer executive functioning
Cognitive Emotion Regulation Questionnaire [28]	CERQ	Describes the frequency with which individuals use specific cognitive strategies (adaptive or maladaptive) to regulate emotions following negative or stressful events	Self‐blame, Other‐blame, Rumination, Catastrophizing, Positive Refocusing, Planning, Positive Reappraisal, Putting into Perspective, Acceptance	1–5 (per item) Higher scores indicate more frequent use of the specific cognitive strategy
Cognitive Flexibility Inventory [27]	CFI	Cognitive flexibility aims to assess an individual's ability to think adaptively in the face of stress	Only the overall score is used.	13–91 (Alternatives) 7–49 (Control) 20–140 (Total) Higher scores indicate greater cognitive flexibility
Generalized Self‐Efficacy Scale [34]	GSES	Self‐efficacy refers to an individual's belief in their own competence to effectively cope with and manage challenging or novel situations	None (unidimensional)	10–40 Higher scores indicate greater self‐efficacy (perceived competence)
Hospital Anxiety and Depression Scale [35]	HAD	Emotional symptoms of anxiety and depression	HAD Anxiety: Emotional symptoms of anxiety HAD Depression: Emotional symptoms of depression	0–21 (per subscale) Higher scores indicate greater anxiety or depression severity
Multidimensional Health Locus of Control (Form C) [29]	HLOC	Locus of health control refers to the extent to which individuals believe their health is controlled by internal factors, chance, powerful others, or medical professionals	Internal: Belief in personal control Doctor: Belief that health or illness is controlled by medical professionals **Note**: Other subscales were not used in this work	1–6 (per subscale) Higher scores indicate stronger belief in the specified locus
Perceived Stress Questionnaire [25]	PSQ‐20	Perceived stress refers to an individual's subjective evaluation of how much stress they experience in their life, reflecting different feelings in response to external demands or challenges	Only the overall score is used	1–4 Higher overall scores indicate greater perceived stress
Working Alliance Inventory (short form) [32]	WAI	Evaluates the quality of the therapeutic relationship between a client and therapist	None (unidimensional)	4–28 Higher scores indicate greater client‐rated working alliance
Treatment Credibility/Expectancy Questionnaire [30]	TE	Treatment credibility refers to a participant's beliefs about how logical, convincing, and credible a treatment appears. Treatment expectancy measures expectations regarding the degree of personal improvement patients anticipate from the treatment	Credibility (belief in logic and plausibility of treatment), Expectancy (belief in personal improvement)	3–27 (for each sub‐scale) Higher scores reflect greater perceived credibility and expectancy regarding the treatment

### 2.4 Statistical Analysis

Quantitative measures are reported as mean and standard deviation (SD), while qualitative (categorical) measures are reported as count and percentage. Changes with treatment in outcome measures (Table 2) are evaluated via linear mixed models with formal statistical inference via the nonparametric bootstrap using 2000 bootstrap samples, due to non‐normality. Correlation between change in patient outcomes and change in psychological measures was evaluated univariately via Spearman rank correlations (Tables 3 and 4) and via multiple linear regression to identify statistically independent correlates of change in patient outcomes (Table 5). Regression models are reported as both unstandardized and standardized regression coefficients, 95% confidence interval (CI), two‐tailed *p*‐value, and explained variance (R^2^) in the change in the patient outcome variable. The standardized regression coefficients are comparable across psychological traits measured on different scales and facilitate the interpretation of the relative magnitude of effects. As this is an exploratory study, no adjustment has been made for the number of hypothesis tests conducted, and the results need to be confirmed in an independent sample.

**TABLE 2:** Change in patient outcome measures during the course of therapy.

	Visit		*d*	*p*
	Baseline	End‐of‐treatment		
Chronic constipation				
N	71	66		
PAC‐QoL	2.2 (0.8) 56	1.6 (0.8) 45	−0.75	*
VAS‐Satisfaction	2.8 (2.4) 65	5.6 (2.4) 58	1.17	*
Chg %CBM	33.2 (30.8) 63	52.2 (37.3) 54	0.56	*
CBM/week	3.8 (4.3) 63	4.7 (3.5) 54	0.23	*
VAS‐QoL	7.2 (2.5) 71	5.9 (2.5) 58	−0.52	*
Fecal incontinence				
N	87	87		
FISI	27.6 (12.0) 82	21.3 (13.2) 75	−0.50	*
FI‐QoL	2.7 (0.6) 72	3.0 (0.7) 68	0.46	*
Control	3.2 (2.0) 84	5.7 (2.6) 79	1.09	*
VAS‐QoL	7.5 (2.2) 86	5.9 (2.7) 79	−0.65	*

Abbreviations: Entries are mean and (standard deviation). d = cohens d, BM = bowel motions, CBM = complete spontaneous bowel motions, QoL = PAC‐QoL, FISI = fecal incontinence severity index, VAS = visual analogue scale (1–10).

* Difference from baseline *p* < 0.05.

**TABLE 3:** Change in psychological constructs during the course of therapy.

Visit	Chronic constipation		smd	*p*	Fecal incontinence		smd	*p*
	Baseline	End‐of‐treatment			Baseline	End‐of‐treatment		
N	71	66			87	87		
HADS: Anxiety	7.5 (4.1) 71	7.2 (2.7) 66	−0.086		5.7 (3.7) 85	6.4 (2.1) 87	0.228	
HADS: Depression	4.8 (3.6) 69	10.1 (3.2) 66	1.244	*	4.1 (3.5) 85	10.4 (2.9) 87	1.398	*
CERQ: Acceptance	3.2 (0.8) 71	2.9 (0.8) 65	−0.297		3.2 (0.8) 85	2.9 (1.0) 85	−0.346	*
CERQ: Rumination	2.8 (0.9) 71	2.3 (0.9) 65	−0.520	*	2.8 (1.0) 85	2.3 (0.9) 85	−0.517	*
CERQ: Positive	3.0 (1.0) 71	3.5 (1.1) 65	0.443	*	2.9 (1.0) 85	3.3 (1.0) 85	0.377	*
CERQ: Positive reappraisal	3.1 (1.0) 71	3.1 (1.2) 65	−0.065		3.0 (1.0) 85	3.0 (1.1) 85	−0.019	
CERQ: Catastrophizing	2.2 (0.9) 71	1.8 (0.8) 65	−0.414	*	2.3 (0.9) 85	2.0 (0.8) 85	−0.381	*
BRIEF: Global	105.8 (20.8) 71	101.8 (21.8) 66	−0.152	*	102.9 (22.0) 87	100.1 (20.5) 86	−0.147	*
PSQ: Overall	2.4 (0.6) 70	2.2 (0.6) 66	−0.260	*	2.2 (0.6) 86	2.1 (0.6) 86	−0.259	*
CFI: Total	109.8 (16.2) 71	111.4 (17.9) 65	0.073		109.2 (16.6) 86	110.3 (17.0) 85	0.052	
HLOC: Internal	3.8 (0.9) 71	4.0 (1.0) 65	0.247		3.7 (0.9) 85	3.9 (1.1) 85	0.176	
HLOC: Doctor	4.2 (0.8) 71	4.2 (1.0) 65	−0.023		4.5 (0.8) 85	4.3 (1.0) 85	−0.192	
GSES: Total	31.9 (4.4) 70	13.2 (1.9) 64	−1.872	*	31.2 (5.0) 83	13.0 (2.0) 82	−1.842	*
TE: Credibility	24.1 (4.5) 70	24.7 (5.4) 65	0.074		25.4 (4.4) 84	26.2 (4.2) 85	0.188	
TE: Expectancy	20.3 (6.4) 70	14.1 (5.4) 65	−0.937	*	22.3 (5.0) 84	15.7 (4.2) 85	−1.167	*

Abbreviations: BRIEF = behavior rating of executive function, CERQ = cognitive emotion regulation questionnaire, CFI = cognitive flexibility inventory, GSES = generalized self‐efficacy scale, HADS = hospital anxiety and depression scale, HLOC = health locus of control, PSQ = perceived stress questionnaire, smd = standardized mean difference, TE = treatment credibility/expectancy.

* *p* < 0.05.

**TABLE 4:** Correlations between change in psychological constructs and change in constipation outcomes from baseline to end of treatment.

Chronic constipation	PAC‐Qol	VAS satisfaction	Chg %CBM	CBM/Week	Chg Qol impact
HADS: Anxiety	−0.033 (0.834) 42	0.101 (0.474) 53	−0.10 (0.946) 53	−0.010 (0.943) 53	−0.206 (0.103) 64
HADS: Depression	0.017 (0.916) 40	0.028 (0.844) 52	−0.17 (0.904) 51	−0.108 (0.449) 51	−0.118 (0.359) 63
CERQ: Acceptance	0.263 (0.092) 42	−0.365 (0.008) 52	0.129 (0.357) 53	0.099 (0.479) 53	0.358 (0.004) 63
CERQ: Rumination	0.357 (0.020) 42	−0.060 (0.673) 52	0.077 (0.585) 53	−0.042 (0.766) 53	0.337 (0.007) 63
CERQ: Positive	−0.157 (0.320) 42	−0.117 (0.411) 52	0.195 (0.163) 53	0.136 (0.330) 53	0.169 (0.184) 63
CERQ: Positive reappraisal	−0.043 (0.788) 42	0.088 (0.533) 52	−0.204 (0.143) 53	−0.076 (0.588) 53	0.068 (0.594) 63
CERQ: Catastrophizing	0.269 (0.085) 42	0.087 (0.538) 52	0.015 (0.918) 53	0.015 (0.918) 53	0.217 (0.088) 63
BRIEF: Global	0.281 (0.071) 42	−0.315 (0.022) 53	0.114 (0.416) 53	0.146 (0.295) 53	0.240 (0.056) 64
PSQ: Overall	0.249 (0.112) 42	−0.259 (0.064) 52	−0.081 (0.568) 52	−0.140 (0.324) 52	0.277 (0.028) 63
CFI: Total	−0.130 (0.411) 42	−0.014 (0.921) 52	−0.134 (0.337) 53	0.045 (0.750) 53	0.131 (0.306) 63
HLOC: Internal	−0.119 (0.454) 42	0.031 (0.829) 52	0.174 (0.212) 53	0.231 (0.096) 53	−0.191 (0.134) 63
HLOC: Doctor	0.153 (0.333) 42	−0.077 (0.588) 52	0.121 (0.389) 53	0.067 (0.634) 53	−0.161 (0.208) 63
GSES: Total	0.135 (0.395) 42	−0.153 (0.289) 50	−0.120 (0.395) 52	0.070 (0.620) 52	−0.011 (0.931) 61
TE: Credibility	−0.114 (0.470) 42	0.297 (0.034) 51	0.002 (0.987) 52	−0.163 (0.249) 52	−0.024 (0.852) 62
TE: Expectancy	−0.287 (0.065) 42	0.257 (0.069) 51	0.075 (0.598) 52	0.064 (0.652) 52	0.038 (0.771) 62

*Note:* b = regression coefficient, all changes are calculated from baseline (visit 1). Correlates are a change in the indicated measure.

Abbreviations: %CBM = percentage of all bowel motions where a sense of complete evacuation was reported, BM = bowel motions, BRIEF = behavior rating of executive function, CBM = complete bowel motions, CERQ = cognitive emotion regulation questionnaire, CFI = cognitive flexibility inventory, GSES = generalized self‐efficacy scale, HADS = hospital anxiety and depression scale, HLOC = health locus of control, PSQ = perceived stress questionnaire, TE = treatment credibility/expectancy, VAS = visual analogue scale.

**TABLE 5:** Correlations between change in psychological constructs and change in fecal incontinence outcomes from baseline to end of treatment.

Fecal incontinence	Chg FISI	Chg FI/QoL impact	Chg VAS/Control	Chg Qol impact
HADS: Anxiety	0.044 (0.717) 70	−0.128 (0.327) 61	0.025 (0.829) 76	−0.081 (0.470) 82
HADS: Depression	−0.025 (0.837) 70	−0.131 (0.313) 61	0.001 (0.992) 76	−0.076 (0.496) 82
CERQ: Acceptance	0.294 (0.013) 70	−0.436 (< 0.001) 63	−0.353 (0.002) 75	0.102 (0.360) 82
CERQ: Rumination	0.159 (0.189) 70	−0.166 (0.192) 63	−0.135 (0.249) 75	0.057 (0.612) 82
CERQ: Positive	0.151 (0.211) 70	0.036 (0.781) 63	−0.087 (0.457) 75	0.240 (0.030) 82
CERQ: Positive reappraisal	−0.116 (0.341) 70	0.343 (0.006) 63	0.329 (0.004) 75	−0.108 (0.336) 82
CERQ: Catastrophising	−0.016 (0.898) 70	−0.127 (0.322) 63	−0.050 (0.668) 75	−0.054 (0.633) 82
BRIEF: Global	0.038 (0.750) 72	−0.295 (0.019) 63	−0.165 (0.152) 77	0.069 (0.536) 84
PSQ: Overall	0.263 (0.027) 71	−0.120 (0.348) 63	−0.230 (0.045) 76	0.200 (0.070) 83
CFI: Total	−0.182 (0.131) 70	0.362 (0.004) 63	0.285 (0.013) 75	−0.004 (0.974) 82
HLOC: Internal	−0.212 (0.078) 70	0.313 (0.013) 63	0.268 (0.020) 75	−0.093 (0.405) 82
HLOC: Doctor	−0.238 (0.047) 70	0.271 (0.032) 63	0.120 (0.306) 75	−0.054 (0.631) 82
GSES: Total	−0.039 (0.754) 67	0.261 (0.044) 60	0.041 (0.735) 71	0.003 (0.982) 77
TE: Credibility	0.037 (0.761) 69	0.066 (0.610) 62	0.313 (0.007) 74	−0.123 (0.274) 81
TE: Expectancy	−0.073 (0.553) 69	−0.107 (0.408) 62	0.134 (0.254) 74	−0.188 (0.093) 81

*Note:* b = regression coefficient, all changes are calculated from baseline (visit 1). Correlates are a change in the indicated measure.

Abbreviations: FI‐QoL = fecal incontinence quality‐of‐life. FISI = fecal incontinence severity index, VAS = visual analogue scale, HADS = hospital anxiety and depression scale, CERQ = cognitive emotion regulation questionnaire, BRIEF = behavior rating of executive function, PSQ = perceived stress questionnaire, CFI = cognitive flexibility inventory, HLOC = health locus of control, GSES = generalized self‐efficacy scale, TE = treatment credibility/expectancy.

## 3 Results

### 3.1 Patients

A total of 135 patients were recruited between June 2016 and December 2018. Of patients in the sample, 71 presented with symptoms of CC (mean age 52, SD 17; 90% female), 87 presented with symptoms of FI (mean age 63, SD 17; 91% female), with these samples including 23 who received overlapping symptoms of both CC and FI. Patients with overlapping CC and FI symptoms were included in both CC and FI analyses. Limited sensitivity analyses indicated that inclusion/exclusion of overlapping patients has little effect on results, and the larger sample size resulting from their inclusion improves the statistical reliability of the data.

### 3.2 Change in Symptom‐Based Patient Outcomes

Among CC patients, there were improvements in all patient‐reported outcomes (Table 2). Patient satisfaction with bowel habit improved substantially with Cohen's d in the large range, while there were moderate/large improvements in PAC‐QoL, VAS‐QoL, and percentage of bowel movement that were complete and spontaneous (Table 2, all *p* < 0.05). The number of complete spontaneous bowel movements in this group increased by an average of 0.9 with biofeedback therapy, a small but clinically significant increase which reached statistical significance (Table 2).

Among FI patients, there was a large increase in patients’ sense of control over bowel habit (d = 1.09, Table 2). Disease impact, as measured by the FISI and VAS QoL (impact), decreased by a moderate amount while FI‐related QoL improved by a moderate amount (Table 2, all *p* < 0.05).

### 3.3 Change in Psychological Measures

A number of psychological traits changed substantially over the course of biofeedback therapy, with the magnitude and direction of changes being similar across CC and FI groups (Table 3). SMD values > 0.4 are considered moderate and > 0.8 as large, whether positive or negative [36]. Measures whose direction of change indicated improvement include reduced use of rumination (repetitive thoughts), reduced catastrophizing, a mild decrease in the acceptance of emotion regulation strategies, a mild decrease in perceived stress, and an increased use of positive strategies in emotion regulation. However, some measures changed in a counterintuitive direction, including an increase in depressive symptoms and large decreases in generalized self‐efficacy and treatment expectancy.

### 3.4 Correlation Between Change in Psychological Measures and Change in Outcomes During Biofeedback Therapy (Tables 4 and 5)

**FIGURE 1:**
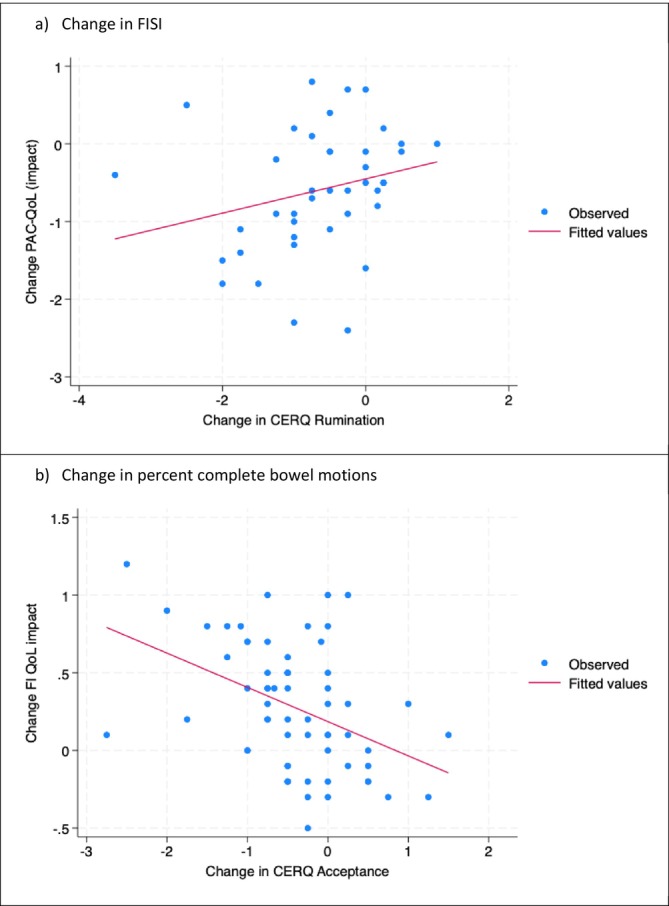
Change in patient outcome correlated with change in psychological measures to illustrate findings in Tables 4 and 5. CERQ, Cognitive emotion regulation questionnaire; FI, Quality‐of‐life; QoL, Quality‐of‐life; PAC, Patient assessment of constipation.

Among patients with CC, there were consistent findings of psychological correlates of change in reported negative impacts on quality of life across the validated PAC‐QoL and the VAS impact on QoL (Table 4). Worsened negative impacts on QoL were associated with increased use of acceptance, rumination, or catastrophizing in emotion regulation (all *r* = 0.26 to 0.36, Table 4). The same was found when perceived stress increased or when overall executive dysfunction (disordered thoughts) increased (*r* = 0.25 and 0.28, respectively, Table 4). Diminished negative impacts on QoL were associated with an increased treatment expectancy over the course of therapy (r = −0.29). Psychological correlates of improved satisfaction with bowel habit were the same as per QoL impact, with the exceptions of the inclusion of increased treatment credibility being associated with increased satisfaction (*r* = 0.30) and change in catastrophizing not being associated with change in satisfaction (r = 0.09). Change in %CBM was not associated with change in any psychological construct (all *r* < 0.2); however, CBM/week was positively associated with an increase in internal locus of control (*r* = 0.23). A scatterplot illustrating an example association between change in CERQ rumination and concurrent change in PAC‐QoL is provided in Figure 1a.

Among patients with FI (Table 5), improved FISI scores during therapy correlated positively with increased use of acceptance in emotion regulation (*r* = 0.29) and an increase in perceived stress (*r* = 0.26). Improved FI QoL was associated with less use of acceptance (r = −0.44) but increased use of positive reappraisal (*r* = 0.34) in emotion regulation, improved executive function, increased cognitive flexibility, internal or doctor locus of control, and general self‐efficacy (all *r* = 0.26 to 0.36). Change in patient‐reported VAS‐control during therapy largely correlated with the same psychological constructs as FI‐QoL (Table 5), and change during therapy in these two constructs was positively correlated (*r* = 0.46). A scatterplot illustrating an example association between change in CERQ acceptance and concurrent change in FI‐QoL is provided in Figure 1b.

### 3.5 Multivariate Models of Change in Outcome (Table 6)

**TABLE 6:** Multivariate models of change in patient outcomes.

Chronic constipation	Slope^A^	Std error	*p*	CI	Slope^B^
VAS‐Satisfaction					
CERQ acceptance	−1.243	0.381	0.001	[−1.989, −0.497]	−0.360
TE credibility	0.268	0.084	0.001	[0.103, 0.433]	0.431
VAS‐QoL					
PSQ overall	3.163	1.445	0.029	[0.331, 5.995]	0.332
CFI total	0.073	0.025	0.004	[0.023, 0.123]	0.310
CERQ acceptance	1.534	0.349	< 0.001	[0.851, 2.218]	0.411
CERQ positive reappraisal	1.206	0.485	0.013	[0.255, 2.158]	0.338
HLOC doctor	−1.640	0.425	< 0.001	[−2.473, −0.808]	−0.425
Fecal incontinence					
FI‐QoL					
BRIEF global	−0.009	0.003	0.006	[−0.015, −0.002]	−0.249
CFI total	0.013	0.004	0.002	[0.005, 0.022]	0.332
HLOC internal	0.137	0.048	0.005	[0.042, 0.232]	0.293

Abbreviations: BRIEF = behavior rating of executive function, CERQ = cognitive emotion regulation questionnaire, CFI = cognitive flexibility inventory, FI‐QoL = fecal incontinence quality‐of‐life, HLOC = health locus of control, TE = treatment credibility/expectancy, VAS = visual analogue scale.

^A^ Unstandardized slope, in units of the outcome measure.

^B^ Standardized slope can be compared across measures.

Among CC patients, decreased use of acceptance in emotion regulation over therapy and increased treatment credibility (positive) over therapy were independent and statistically significant correlates of change in VAS satisfaction, jointly explaining 33% of the variance in change in satisfaction, a large amount (Table 6) with similar standardized effect sizes. An increase in perceived stress, cognitive flexibility, and use of acceptance and positive reappraisal in emotion regulation (all positive), as well as doctor locus of control (negative), were independent and statistically significant correlates of change in VAS QoL, jointly explaining 52% of the variance in change in QoL (Table 6) with standardized effect sizes ranging from 0.332 to (−)0.425.

Among FI patients, increased cognitive dysfunction (negative association), increased cognitive flexibility, and increased internal locus of control (both positive associations) were independent and statistically significant correlates of change in FI QoL, jointly explaining 31% of the variance in change in FI QoL, also a large amount (Table 5), with standardized effect sizes ranging from (−)0.25 to 0.33.

## 4 Discussion

The principles on which anorectal biofeedback teaches patients with defecatory control challenges, such as CC or FI, are well documented [37, 38]. However, while the biofeedback input and clinical outcomes can be observed, the intermediate process by which patients get from input to output is less well understood. Drawing on the psychological therapy literature, this study hypothesized that psychological processes could be involved, such as cognitive function, emotion regulation strategies, and aspects of locus of control. As we could not specify a priori which particular constructs would be most important, we canvassed a range of possibilities and regard this as an exploratory study.

More inclusive entry criteria for constipation were employed in this study rather than the strict Rome IV criteria for functional defecatory disorder (FDD) because it is now recognized that a large proportion of constipation patients that respond to biofeedback are missed by the Rome IV criteria due to restrictions such as requiring the “absence of loose stools” or exclusion of irritable bowel syndrome (IBS) subtypes other than constipation predominant irritable bowel syndrome [39]. Further, we have shown in previous studies [40] that patients not meeting the Rome physiology criteria for FDD still benefit from biofeedback therapy.

For patients undertaking biofeedback therapy for CC, two features of the data stand out that are readily actionable. One is that patients who reported increased use of maladaptive emotion regulation strategies and increased stress also reported decreased QoL over the course of therapy. Psychological therapy as an adjunct may be able to intervene to improve these experiences. The second actionable finding was that improved patient expectancy of the efficacy of treatment improved their satisfaction with bowel habit and reported QoL over the course of therapy, although we acknowledge that the causal direction of these associations cannot be determined by these data. Expectancy has been demonstrated to be modifiable through classical conditioning [41], which is readily implemented. A third clinically interesting, although not actionable, finding is that there was minimal evidence that changes in psychological traits were associated with concurrent changes in recorded bowel habit. There was only one substantive correlation between increased internal locus of control and increased CBM/week, although this did not reach statistical significance. Hence, the data suggest that changes in patients' psychological profile during biofeedback therapy are associated with concurrent changes in patients' perceived well‐being but not with changes in objective bowel function.

Some differences in the pattern of correlations between change in psychological and patient‐reported outcomes were identified in patients receiving biofeedback therapy for FI. In common with the CC findings, increased use of acceptance as an emotion regulation strategy was associated with less improvement in disease severity (FISI), QoL, and control over bowel habit. However, increased use of positive reappraisal in emotion regulation was positively associated with improved QoL and sense of control.

Further, the multivariate models reported in Table 5 suggest that combinations of the changes in psychological traits explain a substantial fraction of the concurrent change in QoL (for both CC and FI) and for satisfaction with bowel habit (CC only). This is important as it suggests that changes in psychological traits during biofeedback therapy are not merely correlates of minimal clinical importance but are potentially major factors in how patients feel their wellbeing is improved by engaging in therapy.

The practical implications of these findings are considerable for the implementation of biofeedback therapy not only within anorectal disorders but potentially extending to the multitude of other DGBI. Our substantial findings are that the efficacy of biofeedback therapy is related to improvements in a number of modifiable psychological traits, which in turn could be more specifically targeted during therapy to maximize treatment efficacy, either by existing clinical staff or by the inclusion of a psychologist in the therapeutic team. It is possible that some of the processes identified in this study as salient are already being directly or indirectly targeted in biofeedback therapy programs, such as attentional control (executive function) or cognitive flexibility, but this is likely to be inconsistent and dependent on happenstance observations of individual clinicians or teams. The findings of this study suggest that purposeful targeting of cognitive processes, emotion regulation strategies, and self‐efficacy may enhance the benefits already seen by many patients and enhance the efficacy of therapy overall. It is also possible that in doing so, the number of biofeedback sessions needed for optimal outcomes could be reduced, making better use of scarce health care resources, as our group has demonstrated [23].

While we believe this study adds useful information to the clinical implementation of biofeedback in disordered defecation (CC or FI), there are also important caveats to be made. An important general caveat is that causal claims cannot be made from a non‐randomized, uncontrolled study design, and we have been careful to avoid making such claims. While this study correlates the change in psychological process with clinical outcomes and speculates on possible mechanisms involved in biofeedback therapy, it is also possible that the symptom changes experienced by patients subsequently bring about changes in constructs such as perceived stress and locus of control. Another is that we chose a range of psychological constructs on the basis of a careful literature review, but this did involve making extrapolations from other conditions. It is possible that there are other constructs more directly involved in the path from therapeutic inputs to patient outcomes that we were unaware of. Finally, and crucially, the findings of this study need replication as the possibility of statistical type I error cannot be ruled out. That said, the majority of the observed correlations between changes in psychological constructs and patient outcomes are logical and consistent with prior expectations. A final important point of context for this work is that individuals suffering from fecal incontinence may be reluctant to disclose their condition and seek health care [13], and that there is much overlap with many patients suffering both constipation and fecal incontinence. Despite this and the fact that biofeedback protocols are often very similar for both conditions, grouping all patients together psychologically could potentially limit our ability to find differences between groups.

Although our data do not directly indicate whether changes in psychological traits cause subsequent changes in patient‐reported outcomes, as opposed to the reverse, the weight of prior evidence of the positive health impacts of adaptive emotion regulation strategies, reducing perceived stress and internal locus of control, strongly suggests that intervention in these areas would enhance the patient outcome of biofeedback therapy.

In summary, given that anorectal biofeedback is thought to be predominantly a learning program, common sense would have made it seem unlikely that biofeedback therapy would cause improvements in patient outcomes without being mediated by some form of central processes. Our hypothesis was that a number of psychological processes would be involved in the response to biofeedback therapy, including cognitive processes. The strong findings of this study support that hypothesis and yield data that could be used to enhance patient engagement and improve the efficacy of biofeedback therapy. The results of our study may induce a quantum shift away from focusing on physiological processes alone to being inclusive and cognizant of the likely major role that psychological factors have to play in the optimization of anorectal biofeedback for CC and FI, and indeed may also play a role in a number of behavioral therapies for other DGBI.

## Author Contributions

**Michael P. Jones:** co‐conceived idea for project, co‐designed study, data analysis, lead manuscript writing. **Yoav Mazor:** co‐conceived idea for project, co‐designed study, assisted with data analysis, contributed to writing, critical review of manuscript. **Alissa Beath:** co‐designed study, collected data, data preparation, contributed to writing, critical review of manuscript. **Gillian Prott:** collected data, data preparation, critical review of manuscript. **Carol Sequeira:** collected data, data preparation, critical review of manuscript. **Allison Malcolm:** co‐conceived idea for project, co‐designed study, assisted with data analysis, contributed to writing, critical review of manuscript.

## Conflicts of Interest

The authors declare no conflicts of interest.

## Data Availability

Research data are not shared.
